# A prediction tool for plaque progression based on patient-specific multi-physical modeling

**DOI:** 10.1371/journal.pcbi.1008344

**Published:** 2021-03-29

**Authors:** Jichao Pan, Yan Cai, Liang Wang, Akiko Maehara, Gary S. Mintz, Dalin Tang, Zhiyong Li

**Affiliations:** 1 School of Biological Sciences and Medical Engineering, Southeast University, Nanjing Jiangsu, China; 2 The Cardiovascular Research Foundation, New York, New York, United States of America; 3 Mathematical Sciences Department, Worcester Polytechnic Institute, Massachusetts, United States of America; 4 School of Mechanical, Medical and Process Engineering, Queensland University of Technology, Brisbane, Queensland, Australia; Stanford University, UNITED STATES

## Abstract

Atherosclerotic plaque rupture is responsible for a majority of acute vascular syndromes and this study aims to develop a prediction tool for plaque progression and rupture. Based on the follow-up coronary intravascular ultrasound imaging data, we performed patient-specific multi-physical modeling study on four patients to obtain the evolutional processes of the microenvironment during plaque progression. Four main pathophysiological processes, i.e., lipid deposition, inflammatory response, migration and proliferation of smooth muscle cells (SMCs), and neovascularization were coupled based on the interactions demonstrated by experimental and clinical observations. A scoring table integrating the dynamic microenvironmental indicators with the classical risk index was proposed to differentiate their progression to stable and unstable plaques. The heterogeneity of plaque microenvironment for each patient was demonstrated by the growth curves of the main microenvironmental factors. The possible plaque developments were predicted by incorporating the systematic index with microenvironmental indicators. Five microenvironmental factors (LDL, ox-LDL, MCP-1, SMC, and foam cell) showed significant differences between stable and unstable group (p < 0.01). The inflammatory microenvironments (monocyte and macrophage) had negative correlations with the necrotic core (NC) expansion in the stable group, while very strong positive correlations in unstable group. The inflammatory microenvironment is strongly correlated to the NC expansion in unstable plaques, suggesting that the inflammatory factors may play an important role in the formation of a vulnerable plaque. This prediction tool will improve our understanding of the mechanism of plaque progression and provide a new strategy for early detection and prediction of high-risk plaques.

## Author summary

Besides the traditional systematic factors, the influences of the local microenvironmental factors on atherosclerotic plaque progression have been demonstrated. Mathematical and computational modeling is an important tool to investigate the complex interplay between plaque progression and the microenvironment, and provides a potential way toward the prediction of plaque vulnerability according to the comprehensive evaluation of both morphological and/or biochemical factors in tissue level with microenvironmental factors in cellular level. We performed patient-specific multi-physical modeling study on four patients to obtain the evolutional processes of the microenvironment during plaque progression and predicted the possible plaque developments. A scoring table integrating the dynamic microenvironmental indicators with the classical risk index was proposed to differentiate their progression to stable and unstable plaques. Based on patient-specific imaging data, the mathematical model will provide a novel method to predict the changes of plaque microenvironment and improve ability to access the personal therapeutic strategy for atherosclerotic plaque.

## Introduction

Atherosclerosis is the process in which plaques, consisting of lipids, monocytes, macrophages (MΦs), vascular smooth muscle cells (SMCs) and calcium, are built up in the walls of arteries as a chronic inflammatory response. Coronary atherosclerosis can cause myocardial infarction and heart failure [1]. Although multiple systemic atherosclerotic risk factors have been identified, including age, family history, hypertension, hypercholesterolemia, diabetes and so on, advances in molecular and cellular research have significantly enhanced our understanding of the influence of the local biochemical/biophysical microenvironment on atherosclerotic plaque progression. It is now acknowledged that atherogenesis is a function of both systemic atherosclerotic risk factors and the local microenvironmental stimuli [2,3].

Multiple microenvironmental factors contribute to plaque formation and progression, including arterial mechanics, hemodynamics, matrix composition, lipid deposition, inflammation and neovascularization from vasa vasorum (VV) [2,4–8]. The cellular components (MΦs, SMCs, endothelial cells, etc.) and their extracellular environment exist in a state of dynamic reciprocity, which influences all stages of plaque progression and determines the plaque fate as to whether it would develop to a vulnerable phenotype. For example, in the early phase, hypercholesterolemia conditions increase low-density lipoprotein (LDL) infiltration and retention into the injured endothelial layer in response to disturbed blood flow pattern, leading to the accumulation of inflammatory cells by the release of pro-inflammatory factors, such as monocyte chemoattractant protein (MCP-1) [7,9,10]. Meanwhile, vascular SMCs undergo phenotypic dedifferentiation that from quiescent phenotype to a synthetic and activated phenotype, in response to pro-inflammatory cytokines and oxidized LDL (ox-LDL) [11–13]. In addition, SMCs express a number of pro-inflammatory chemokines in turn and even phenotypically transform to macrophage-like cells [11,12]. Another important pathological process involved in the coupled interactions among the microenvironmental components within the plaque is intraplaque angiogenesis [2], which refers to the disorganized and abnormal neovascularization from adventitial VV in response to the hypoxic microenvironment in the thickening intimal. Intraplaque angiogenesis together with the associated intraplaque hemorrhage (IPH) contributes to the accumulation of inflammatory cells and lipoproteins through the leaky angiogenic microvessels, which are vital characteristics of atheroma microenvironment in vulnerable plaques [2,6]. To summarize, an understanding of plaque development and progression requires the elucidation of multiple factors and their interactions within the microenvironment.

Unfortunately, there are fundamental gaps in our knowledge of the underlying mechanisms that contribute to the influences of plaque microenvironmental factors on atherogenesis in vivo. The main reason for this is that the microenvironment that determines the disease activity, including lipid deposition, inflammation, neovasculature and hemorrhage, cannot be assessed by non-invasive plaque-imaging techniques (such as ultrasound, CT and MR) which are widely used to obtain plaque morphological characteristics in clinic. Although advanced imaging techniques, including intravascular ultrasound (IVUS), optical coherence tomography (OCT) and positron emission tomography–computed tomography (PET/CT), now have permitted more accurate assessment of the plaque composition and even the atherosclerotic disease activity, the limited spatial resolution and lack of dynamic changes of microenvironmental factors give rise to barriers to quantitatively analyze the functional activity in atherosclerotic microenvironments.

Besides the developments of novel imaging techniques, mathematical modeling and numerical simulation have been proven useful for quantitatively assessing the dynamic changes of cellular and acellular components involved in the plaque microenvironment and predicting plaque growth and possible rupture [14,15]. In our previous studies, we have developed a multi-physical mathematical model by coupling lipid deposition, inflammation, neovascularization and intraplaque hemorrhage, to investigate the pathophysiological responses of plaques to dynamic changes in the microenvironment [16,17]. For the first time, the promotion of intraplaque angiogenesis on the accumulation of ox-LDL and macrophages in the plaque lesion and its quantitative contribution to the induction and progression of plaque destabilization were demonstrated by using this multi-physical model. This study is an application of our previously developed model for identifying the spatial-temporal dynamics and progression in coronary atheroma microenvironment based on patient-specific virtual histology(VH)-IVUS imaging data. The microenvironmental components in this model consist of four main parts, i.e., the plaque morphology and composition, the lipid deposition, the inflammation and the intraplaque neovascularization. To assess the personalized plaque microenvironment, simulations on a single patient data set are performed and the predicted changes of plaque composition are validated with the follow-up data from the same patient at the later time points. This study aimed (a) to describe the dynamic quantitative changes of the main factors in plaque microenvironment; (b) to predict the plaque development according to both systemic risk factors and microenvironmental factors; and (c) to investigate the distinct role of lipid and inflammatory microenvironment in the formation of a vulnerable plaque.

## Results

### Dynamic variation of the microenvironmental factors

**Fig 1:**
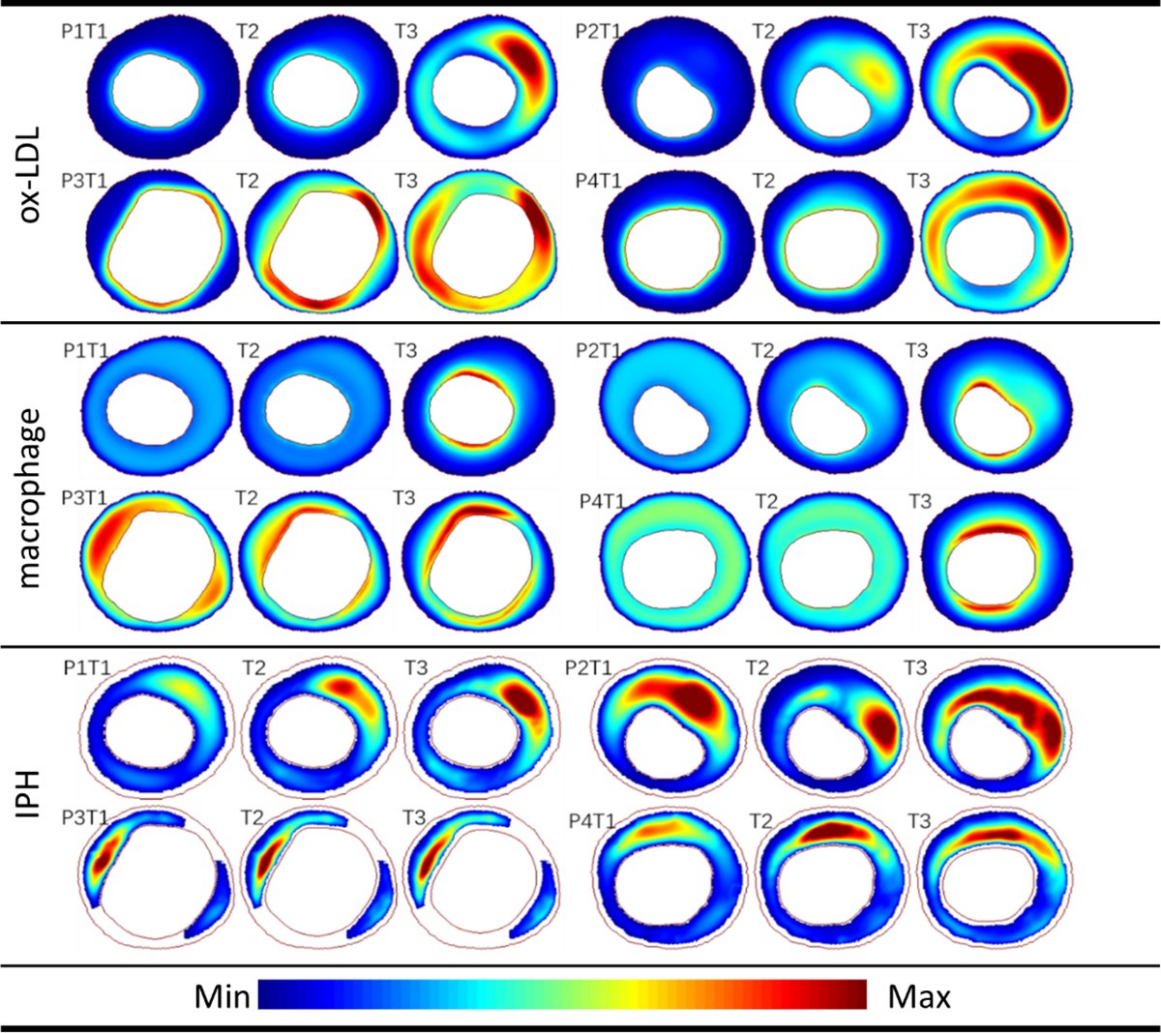
The progression of lipids, inflammation and angiogenesis microenvironment. The three typical factors, i.e., ox-LDL, macrophage and IPH, represent lipid, inflammation and angiogenesis levels in plaque microenvironment. Distributions of ox-LDL, macrophage and IPH at T1, T2 and T3 are shown during the plaque development. The values in all figures of each factor are normalized to the range of 0 to 1. A video of the dynamics of all the variables involved in the simulation is provided in S1 File.

The distribution changes of the three main variables (ox-LDL, MΦs and IPH) in four patients (P1-P4) are displayed in Fig 1, which represent the critical contributors in the lipid, inflammation and angiogenesis microenvironment to atherogenesis. An animation of the dynamics of all the variables involved in the simulation can be found in S1 File. At the early stage of the simulation (T1), most ox-LDL concentrated near the inner intima, while the area of high ox-LDL concentration gradually enlarged in the thickening intima (follow-up, T2) due to the extravasation of lipoprotein from the neovasculature, resulting in the lipid deposition near the outer intima in the plaque (three years after T1, the end of simulation, T3). Similar to the lipid deposition, the exacerbating inflammation were found during plaque progression. A high concentration of IPH was observed in the necrotic core (NC) area throughout the plaque development, especially, at the shoulder of NC in P2.

**Fig 2:**
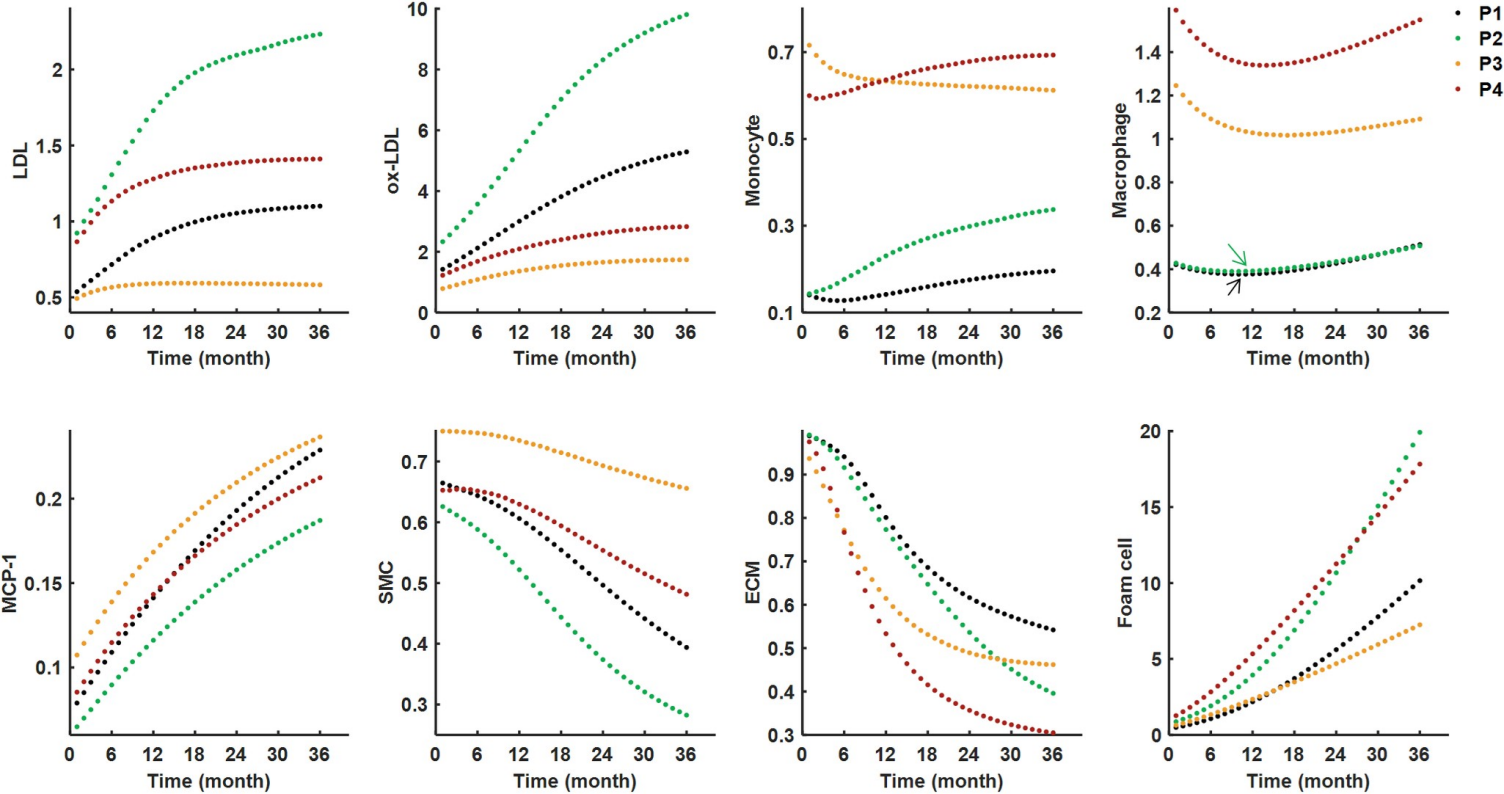
The growth history curves of the average values for the plaque microenvironmental factors. Eight variables represent the development of lipid (LDL, ox-LDL) and inflammatory (monocyte, macrophage and MCP-1) microenvironments and the growth of necrotic core (SMC, ECM and foam cell). The values on the y-axis are dimensionless. The arrows in macrophage panel point to the curves of P1 and P2, which are close to each other.

The heterogeneity of plaque microenvironment for each patient was observed apparently from the growth history curves of the average values of the eight main variables, including LDL, ox-LDL, monocyte, macrophage, MCP-1, SMC, ECM and foam cell (Fig 2). Here, we focused on temporal variation in the lipid (denoted by concentration of LDL and ox-LDL) and inflammatory (denoted by the density of monocytes and macrophages) microenvironments. The reference values of the eight variables can be found in S4 File. Although there were no significant differences among the development trends between the four patients, the variation and the heterogeneity of plaque microenvironment between individuals were remarkable. For example, both a mild lipid deposition and a severe inflammation were found in P3 (yellow dotted line), resulting in an indistinctive apoptosis of SMCs. Nevertheless, an opposite situation happened in P2 (green dotted line), where a high level of lipid and a low level of inflammation yielded a notable decrease in SMCs (>50%).

### Prediction of plaque development

**Fig 3:**
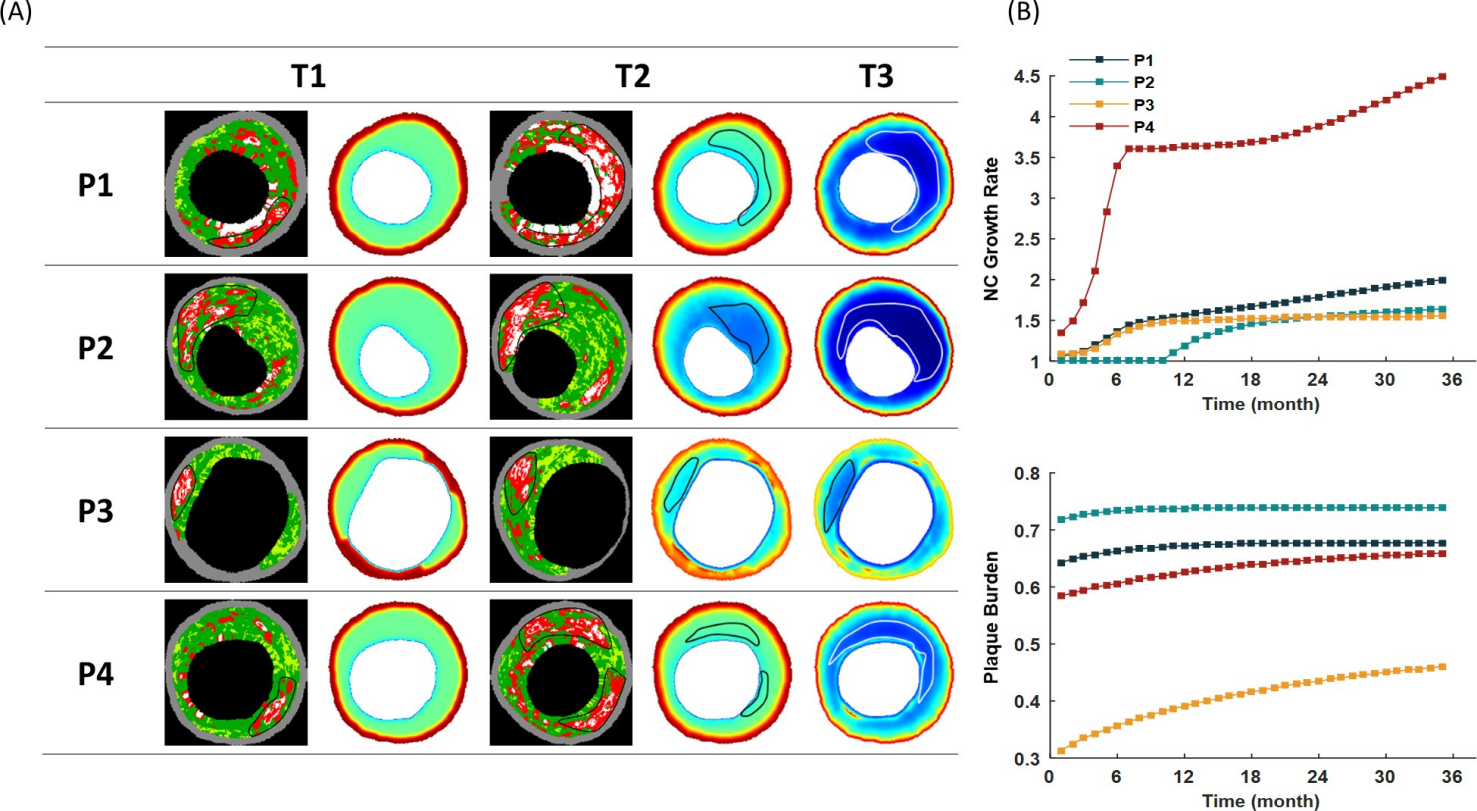
The plaque necrotic core expansion. (A) Comparison of simulation results with VH-IVUS images at T1 and T2. The NC areas are circled by black/white lines in both VH-IVUS images and simulation results. The developments of NC are predicted at T3 based on the simulated dynamics of the plaque microenvironment. (B) The growth history curves of NC area and PB of the patients for three years. The relative values of NC area are compared with the VH-IVUS data at T1.

Fig 3A displays a comparison between the simulation results of the NC areas with the VH-IVUS images. The NC areas at T1 and T2 in the images were delineated based on the image segmentation of VH-IVUS data; while the expanded NC areas at T2 and T3 in the simulation results were determined according to the increasing areas of apoptotic SMCs (NC area was outlined with black/white border in the plaque). Different degrees of increase in plaque area and NC area were found in all four patients. Thin cap fibroatheroma (TCFA) with a large NC was observed especially in P2 and P4 at T3 stage, which suggests that these two plaques may be more likely to become vulnerable plaques. On the contrary, the flaky distributed calcification (white area shown in VH-IVUS) in P1 indicated a stable plaque. In addition, the quantitative curves of the growth rate of NC and plaque burden (PB) for the four patients are provided in Fig 3B, where the relative values of NC area were obtained by comparing to their values shown in the VH-IVUS images at T1. There was a remarkable increase in NC area of P4 at the early stage, while the NC areas in the other three patients increased steadily. Actually, the growth rate of NC in P4 was as high as 1.75 during the follow-up period according to the VH-IVUS images (between T1 and T2). According to the simulation results, the NC area in P4 would increase by 4.55 times after three years (between T1 and T3). An apparent increase (32.6%) of PB was found in P3, while there were little changes (<10%) of PB in the other three patients.

Taking into account of both systematic and microenvironmental factors, we proposed a scoring scale to predict a possible plaque development (Table 1). The scores of PB and LDL were high in P1, suggesting a severe lipid deposition in the plaque. However, the local hemodynamics was not favorable for plaque development (one score for wall shear stress, WSS), resulting in a relatively benign microenvironment in P1. In addition, the flaky distributed calcification areas were shown in the VH-IVUS image (white regions in Fig 3A). Therefore, a stable plaque with macrocalcification was reckoned as its future growing of P1’s plaque. Although the evaluation of the microenvironmental factors in P3 was quite severe, putting together with the other indictors (plaque morphology and blood test) indicated it was a stable plaque. Consequently, the rate of plaque development in P3 was relatively slow, and the NC area was not remarkable during the plaque progression (see Fig 3B). Contrary to the predictions of P1 and P3, both P2 and P4 have the highest scores (Total score = 20), which suggested vulnerable plaques were more likely to be found in these patients. In summary, combing with the analysis of VH-IVUS data and the simulation results, P2 and P4 were classified as unstable group, while P1 and P3 as stable group.

**Table 1:** The multi-factorial scoring scale.

	PB*	EI*	LDL*	HDL*	WSS†	MΦ†	SMC†	ox-LDL†	Total	Predictions for T3
P1	3	2	3	1	1	1	2	2	15	stable plaque with macrocalcification
P2	3	3	3	3	1	1	3	3	20	vulnerable plaque
P3	1	3	1	1	3	3	1	2	15	stable plaque with low growth rate of NC
P4	2	2	3	3	3	3	2	2	20	vulnerable plaque

The predictions for plaque development at T3 are based on the total scores and the component analysis according to VH-IVUS images at T2. The symbols * and † indicate the factor being derived from patients data or simulation results, respectively. PB = plaque burden, EI = eccentric index, LDL = low-density lipoprotein, HDL = high-density lipoprotein, WSS = wall shear stress, MΦ = macrophage, SMC = smooth muscle cell, ox-LDL = oxidized LDL, NC = necrotic core.

### Influence of microenvironmental factors on plaque progression

**Fig 4:**
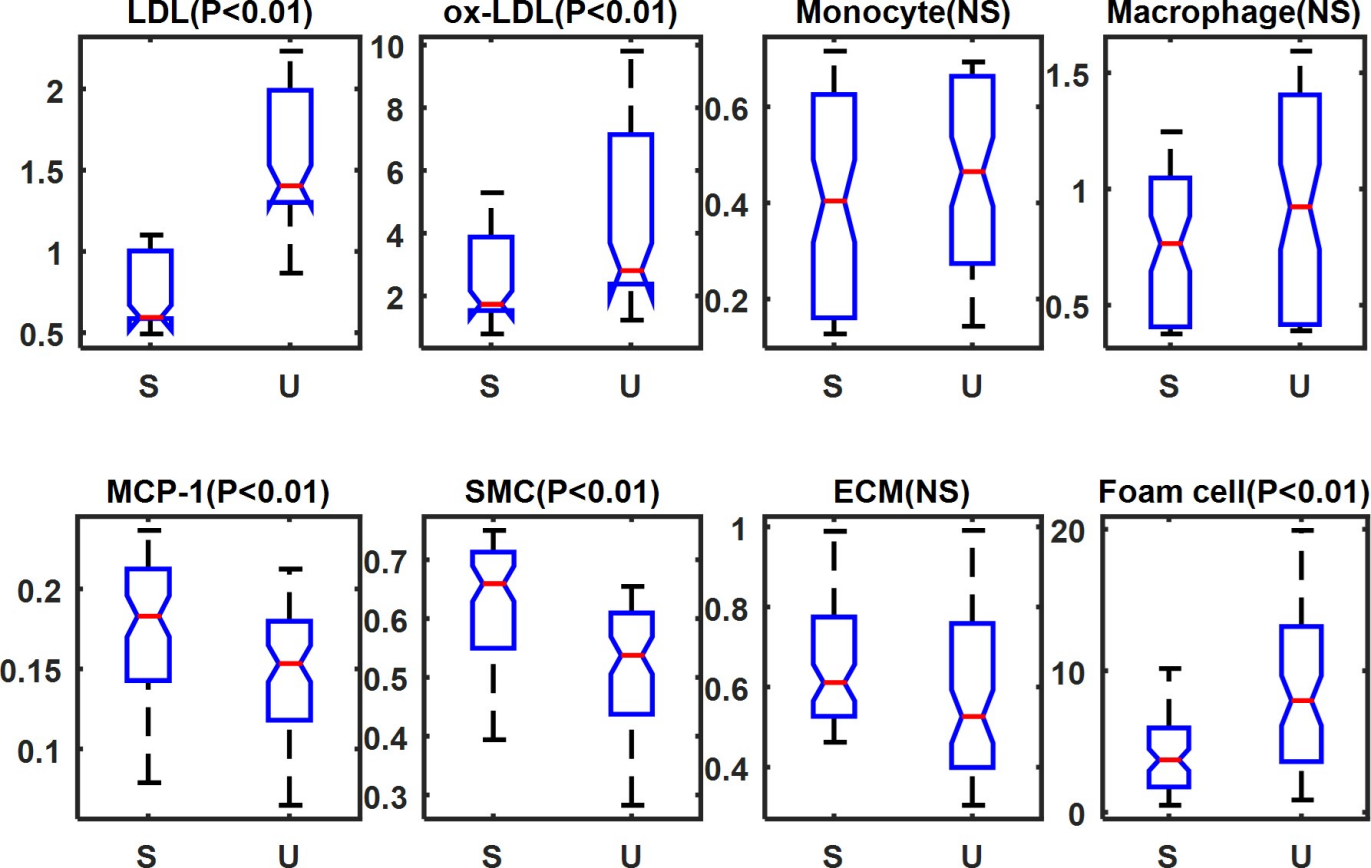
Comparisons of the changes of microenvironmental factors during plaque progression between stable group (S) and unstable group (U). The development of each factors between two groups of patients is compared by independent t-test. In each plot, y-axis values represent the dimensionless concentration of each variable. NS = not significant.

According to the prediction for plaque progression, P1 and P3 were categorized as the stable group, while P2 and P4 as unstable group. Fig 4 shows the difference of the changes of microenvironmental factors during progression between the two groups. Independent t-test demonstrated that there were significant differences in the five microenvironmental factors (LDL, ox-LDL, MCP-1, SMC, and foam cell, P< 0.01). However, no significant differences were found in monocytes (P = 0.11), macrophages (P = 0.011), and ECM (P = 0.02).

Then, we investigated the principal factors that influenced the NC growth of the two groups by using the Spearman’s correlation analysis. As shown in Table 2, in the stable group, LDL, ox-LDL, SMC, and foam cell showed strong corrections with the NC growth (r = 0.7827, 0.8810, -0.8361, 0.8566 respectively). While in the unstable group, monocyte, macrophages, MCP-1, and ECM showed strong correlations with the NC growth (r = 0.9919, 0.8692, 0.7797, -0.8406, respectively). It was noteworthy that the inflammatory microenvironment (monocyte and macrophage) had a significant negative correlation with NC expansion in the stable group (r = -0.3389 and r = -0.3076, respectively). In contrast, it had a very strong positive correlation in the unstable group (r = 0.9919 and r = 0.8692, respectively). This result indicated that the inflammatory factors could be beneficial in stable plaque while detrimental in unstable plaque, due to its interactions with other microenvironmental factors.

**Table 2:** The correlation between microenvironmental variables and NC growth rate in different groups.

	Stable Group		Unstable Group	
**variables**	r	p-value	r	p-value
**LDL**	0.7827*	<0.01	-0.1346	0.2596
**ox-LDL**	0.8810*	<0.01	-0.3925	<0.01
**Monocyte**	-0.3389	<0.01	0.9919*	<0.01
**Macrophage**	-0.3076	<0.01	0.8692*	<0.01
**MCP-1**	0.6637	<0.01	0.7797*	<0.01
**SMC**	-0.8361*	<0.01	0.0850	0.4779
**ECM**	-0.4421	<0.01	-0.8406*	<0.01
**Foam cell**	0.8566*	<0.01	0.5808	<0.01

* indicates the variable shows the strong correlation with plaque progression.

## Discussion

Although the formation and progression of atherosclerotic plaques is understood to be mainly driven by the systemic factors, both in vitro and in vivo observations over the past several decades have established that plaque development involves a complex coordination of systemic and local microenvironmental factors that determine how plaques progress [2,18,19]. Uncovering the underlying mechanisms and crosstalk of plaque microenvironment constitutes as prerequisite for the exploration of possibilities targeting the processes within the plaque microenvironment as novel therapeutic strategies. Recently, the results of the Canakinumab Anti-inflammatory Thrombosis Outcome Study (CANTOS, targeting the interleukin-1β) trial show conclusive proof that reduction of inflammation by inhibiting the IL-1β pathway activation can significantly lower coronary artery disease morbidity and mortality [20]. However, there is currently a paucity of data on the dynamic interactions of the plaque microenvironmental components, due to the unavailability of imaging techniques to quantitatively investigate the changes of plaque microenvironment in both animal experiments and clinical settings. In this context, mathematical modeling provides a potential way to assess the dynamics of the plaque microenvironment by describing the spatial-temporal changes of key cells and chemicals according to physical principles and known pathophysiological interactions. To this end, we performed the evolutional simulations of plaque microenvironmental dynamics based on patient follow-up data and predicted the development of microenvironmental factors as well as plaque phenotype in patient-specific atheroma on theoretical and quantitative grounds.

With the information of plaque microenvironment as initial input, including plaque morphology and composition obtained by imaging technique and biochemical indicators by blood examination, the dynamics and development of the main microenvironmental factors can be calculated for next three years by using the developed model system. Based on these results, the evolutional progress of a specific plaque can be predicted. The evaluation indicators of plaque microenvironment include lipid deposition, inflammation, IPH and apoptosis of SMCs. The results clearly demonstrate the heterogeneity of the microenvironmental factors within plaque and during the plaque progression. It has been suggested that plaque progression is a modifiable step in the evolution of atherosclerotic plaque [21]. Therefore, a dynamic and quantitative description of plaque microenvironment can provide direct information for personalized treatment to improve the long-term outcomes. In addition, a scoring scale table for each patient is presented, which comprises the dynamic microenvironmental indicators with the classical risk index (morphological and biochemical index). For the very first time, a quantitative evaluation is proposed by a comprehensive consideration of both morphological factors in tissue level and functional factors in cellular level. We believe the improved evaluation system can help us better understand the interactions among plaque microenvironmental factors and may allow us to predict a possible development of a plaque on an individual basis.

According to the prediction for the plaque progression, the patients were divided into two groups, i.e., stable and unstable group. We investigated the differences of the main microenvironmental factors between the two groups. The statistical analysis revealed that the lipid components (LDL and ox-LDL) rather than the inflammatory factors (monocyte and macrophage) exhibited significant differences between the stable plaques and the unstable ones. In addition, the correlation test of the microenvironmental factors with NC enlargement in different groups was performed. In the stable group, the lipid components had a strong positive correlation with the NC expansion, while in the unstable group, the inflammatory factors that had a very strong positive correlation with the NC expansion. This interesting result may be used to explain why not all clinical trials provided a beneficial cardiovascular effect especially in the anti-inflammatory therapy [22]. This also suggests that the patients who would better benefit from these therapies (such as the unstable group in this study) could be identified according to the dynamics of their plaque microenvironment. Although the correlation analyses of these microenvironmental factors with clinical outcomes still need to be validated by more clinical studies, the present results emphasize the heterogeneity of plaque microenvironment between individuals and its complex role in plaque development, and provide a potential path toward the investigation of an improved and targeted atherosclerosis therapy.

Several imaging modalities are currently used in vivo to characterize one or more plaque microenvironmental factors. For instance, dynamic contrast-enhanced MRI (DCE-MRI) is proposed to study the intraplaque microvasculature quantitatively and to test the relationship between adventitial perfusion and IPH, while ^18^F-FDG PET-CT, a noninvasive functional imaging technique, is widely used to evaluate plaque inflammation by macrophage-targeted agents [23,24]. Compared with the conventional structural imaging techniques identifying the site and severity of luminal stenosis, these functional assessments may provide more informative values in studying the dynamic microenvironment and consequently to evaluate plaque vulnerability. However, it is unrealistic to carry out multi-modality imaging for every patient due to the technological and socio-economic issues. In this context, this proof-of-concept study aims to present a novel personalized evaluation for coronary atherosclerotic plaque microenvironment by incorporating a generalized mathematical modeling system with patient-specific clinical data. The power of mathematical modeling lies in its ability to reveal the underlying dynamic mechanism and the physical principles that might have been overlooked in previous traditional studies. At its best, mathematical modeling provides quantitative supplementary for the imaging data, and enables us to make predictions and early identify which plaque rupture is likely to occur, and leads to a novel and improved ability to assess plaque vulnerability. This will allow actions to be taken in a timely manner to reduce risk of eventual fatal events on an individual basis. Although we have demonstrated the simulation based on VH-IVUS images in this study, further research should be addressed by incorporating other available imaging data to expand the application of this mathematical model in clinical assessment.

There are several limitations in this study. First, the predicted plaque progression should be validated by clinical outcomes and more patients’ data should be included to demonstrate the statistical significance of this study. Extensive large-scale patient study will be needed to validate the model before it can be used as a prediction tool in clinic. Second, the current model excluded the calcification that is associated with the apoptosis of macrophages and SMCs, and interacts with other microenvironmental factors such as inflammation. Third, the scoring system was determined by comparing these four patients to obtain the relative value of the severity, which may be only applicable to the patients in this study. In addition, two-dimensional simulations predict only the local progression of plaque development, making it difficult to evaluate vascular lesions. Three-dimensional modeling with more patient-specific studies and addition of other potential factors will make the system more robust and support in prediction of plaque development. Finally, the balance between pro-atherogenic factors and anti-atherogenic factors in plaque microenvironment was not investigated. Considering the dynamic balance among multiple microenvironmental factors was a major study in itself and its influence on plaque development should be addressed in future work.

In conclusion, an image-based patient-specific multi-physical model is developed which can simulate the spatial-temporal evolution of plaque progression as well as the dynamic variations of plaque microenvironment. This enables us to make predictions and early identify the high-risk rupture-prone plaques, leads to a novel, improved ability to assess plaque vulnerability, and allows actions to be taken in a timely manner to reduce risk of eventual fatal events on an individual basis. It is found that the inflammatory microenvironment has a negative correlation with NC expansion in stable plaques while it has a very strong positive correlation in unstable plaques, suggesting that inflammation may be beneficial or detrimental during plaque progression, depending on the interactions with other microenvironmental factors.

## Methods

### Ethics statement

Patient follow-up IVUS data of coronary plaques were acquired from four patients at Cardiovascular Research Foundation (New York, NY). The Institutional Review Board (IRB) at Cardiovascular Research Foundation approved the protocol. All patients provided written informed consent.

### Study design

**Fig 5:**
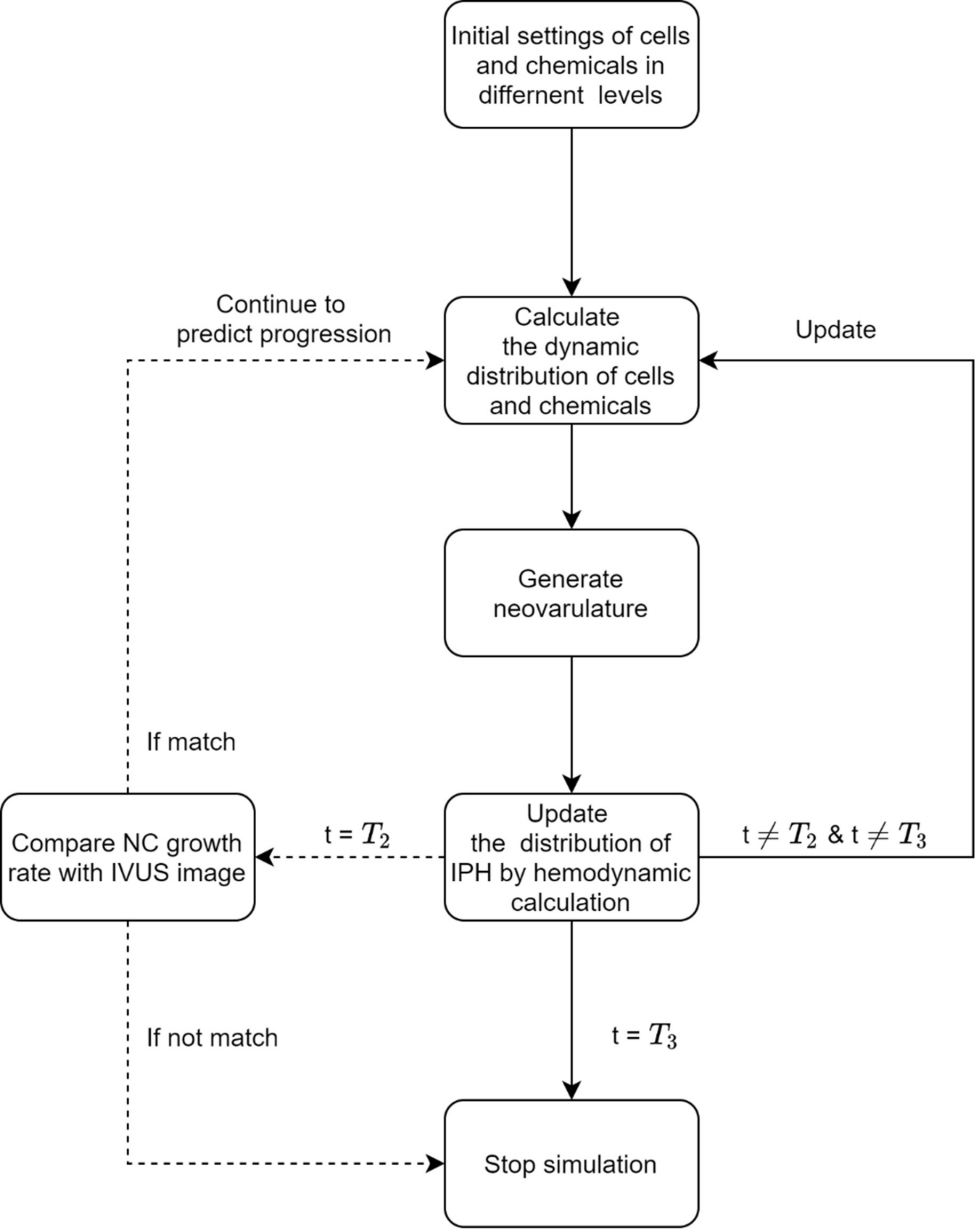
Schematic diagram for simulation algorithms.

The proposed simulation system is described in Fig 5, in which the whole process consists of two steps: validation and prediction. Since the initial inflammation and neovascularization in the plaque microenvironment cannot be assessed by IVUS imaging, we first identified the initial microenvironmental factors at baseline (T1) images, by comparing the simulation results calculated from different levels of macrophages concentration and microvascular density with follow-up images at T2. The quantitative results of comparison were provided in S2 File. Once the plaque microenvironment for each patient at T1 was determined, the simulation was performed to assess the dynamic changes of the main cellular and acellular components involved in the plaque progression. The prediction was then conducted to obtain the plaque development at the end of simulation, T3 (three years after T1).

The major assumptions in this model were listed below:

1. Three different levels of microvessels density and inflammation inside the plaque were set to be initial conditions to carry out three simulations respectively. Intraplaque neovascularization had positive correlation with inflammation in the plaque. The status of intraplaque angiogenesis and inflammation can be validated by comparing the growth rate of NC at T2 stage with IVUS data.
2. The VH-IVUS slice with the maximum PB at T1 was set to be the simulation region and provide the corresponding plaque composition as the initial conditions. The transport and interactions of all variables were restricted within the 2D slice. There was no mass transport at the outer boundary (i.e., external elastic membrane).
3. The interaction relationship among the variables involved in the model system would not change during the plaque progression.
4. Based on the CFD calculation of fluid-structure interaction (FSI) models at T1 and T2, the change of WSS was 11.11%, 8.00%, 1.09% and 18.06% for P1, P2, P3 and P4, respectively. Its effect on LDL deposition was very small and negligible. Therefore, the WSS was assumed to be remaining to the value at T1 stage.
5. The different phenotype of macrophages, such as M1 and M2, and their distinct influence on the inflammatory response of plaque, were not under consideration in the current model.
6. The NC area in simulation was defined as the results of SMC apoptosis in the thickening intima.

### Data acquisition

Follow-up IVUS with virtual histology imaging data of coronary plaques were obtained at Cardiovascular Research Foundation (New York, NY), from 4 patients (3Males, 1Female) who had percutaneous coronary intervention (PCI). The IVUS follow-up was at about 6 months or one year. A synthetic-aperture-array, 20-MHz, 3.2-French catheter (Eagle Eye, In-Vision Gold, Volcano) with motorized catheter pullback (0.5 mm per second) was used to acquire IVUS data.

Level of serum creatinine, fasting lipids, glucose, glycated hemoglobin, and high-sensitivity C-reactive protein were measured at baseline. Fusion of IVUS data and X-ray angiography to reconstruct 3D arterial geometry were performed after the segmentation and co-registration of the one-by-one paired slices at baseline (T1) and follow-up (T2) images. IVUS-based 3D FSI models with cyclic bending were constructed for each coronary to assess the flow WSS conditions (S3 File). For each patient, the paired slices with the maximum plaque burden/area were selected in this study. The detailed demographical information of patients was listed in Table 3.

**Table 3:** Patient demographical information.

Patient ID	P1	P2	P3	P4
**Gender**	Male	Female	Male	Male
**Age**	52	42	71	49
**Vessel**	RCA	RCA	LCX	unknown
**Systolic BP**	113	110	171	134
**Diastolic BP**	73	66	95	76
**Weight (kg)**	95.26	86.64	109	100
**Height(cm)**	178	175	178	172
**BMI**	30.06	28.21	34.40	33.80
**Diagnosis history**	None	None	Unknown	Unstable Angina
**Total Cholesterol (mg/dL)**	210	203	132	188
**HDL (mg/dL)**	38	19	31	19
**LDL (mg/dL)**	138.4	157.8	70.4	144.4
**Triglycerides (mg/dL)**	168	131	153	123
**Insulin(mcU/mL)**	19.4	55	11	9
**HbA1c**	5.9	5.8	6.6	4.8
**Smoke**	Yes	Yes	No	Yes
**WSS (dyn/cm**^**2**^**)**	140.07	129.9	85.8	77.11
**Time interval (month)**	7	13	12	5

All data are obtained at the T1 stage. Vessel is the taken vessel in the model for each patient, and the time interval is the interval from T1 to T2. RCA = right coronary artery, LCX = left circumflex coronary artery.

### Image-based patient-specific multi-physical modeling

**Fig 6:**
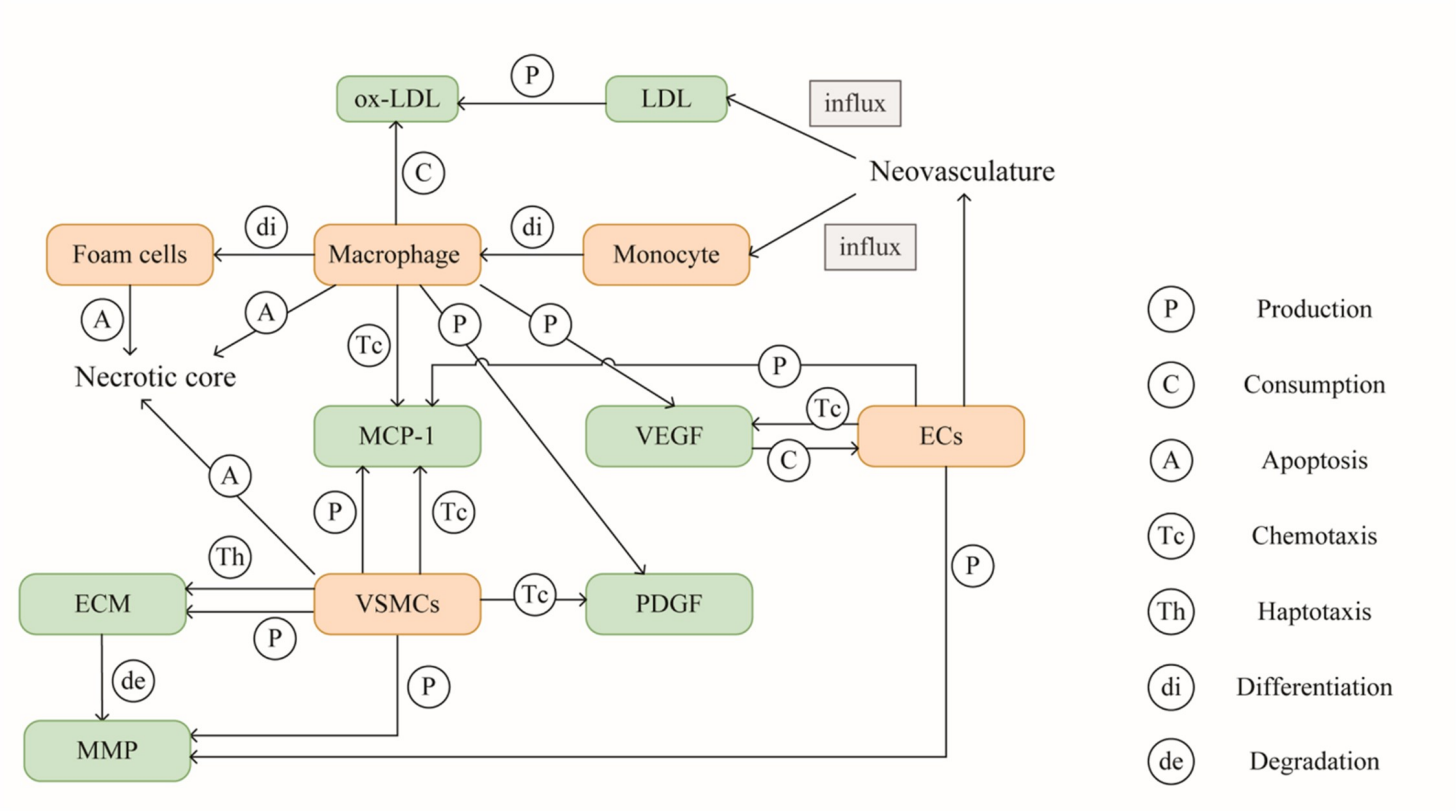
The interactions between variables in the model. Seven pathophysiological progresses (circle) among cellular (yellow box) and acellular (green box) factors are included in the model, and every arrow represents a reaction term modelled in the reaction-diffusion equations.

Based on our recently developed model [16,17], we modeled plaque microenvironment as a continuum medium that the cellular/acellular factors diffuse within the thickening intima as well as interact with each other. In particular, we considered four main pathophysiological processes during plaque development, i.e., lipid deposition, inflammatory response, migration and proliferation of SMCs, and neovascularization. These four pathophysiological phenomena were coupled based on the following experimental and clinical observations: (a) ox-LDL activates the expression of proinflammatory cytokine such as MCP-1 to facilitate the recruitment of more monocytes into the lesion; (b) the neovasculature provides a potential way for LDL and monocytes into the intima by extravasation from the leaky vessel wall; (c) the accumulation of lipoprotein and inflammatory cells (monocytes and macrophages) promotes SMCs migration and proliferation, resulting in a hypoxic microenvironment to induce further angiogenesis in the thickening intima. We modeled the cellular and acellular components involved in the above four processes. Namely, the cellular components consist of endothelial cells (ECs), macrophages, monocytes, foam cells and SMCs, while the acellular counterparts include LDL, ox-LDL, MCP-1, VEGF, MMP, ECM and extravascular plasma concentration. The interactions between all variables in this model are illustrated in Fig 6. The dynamics of these twelve variations were described by coupled reaction-diffusion equations as follow:

$$\frac{\partial C_{i}}{\partial t}=\overset{\mathrm{Diffusion}}{\overset{︷}{D_{C_{i}}\nabla^{2}C_{i}}}+\overset{\mathrm{Production}}{\overset{︷}{\lambda_{P}C_{j}}}-\overset{\mathrm{Consumption}}{\overset{︷}{\lambda_{C}C_{j}}}-\overset{\mathrm{Apoptosis}}{\overset{︷}{\lambda_{A}C_{i}}}-\overset{\mathrm{Chemotaxis}}{\overset{︷}{\nabla (\lambda_{Tc}C_{i}\nabla C_{j})}}-\overset{\mathrm{Haptotaxis}}{\overset{︷}{\nabla (\lambda_{Th}C_{i}\nabla C_{j})}}-\overset{\mathrm{Degradation}}{\overset{︷}{\lambda_{de}C_{i}C_{j}}}$$ (1)

where *C*_*i*_ denoted one plaque microenvironmental factor, and the first terms on the right-hand side of the equation described the diffusion of *C*_*i*_ with diffusion coefficient $D_{C_{i}}$. The reaction terms of *C*_*j*_ were modeled based on the proved pathophysiological knowledge during the plaque progression, including the production, consumption, chemotaxis, haptotaxis, and differentiation by other microenvironmental factor *C*_*i*_, as well as the apoptosis by itself. The detailed explanations of reaction terms involved in the model system are listed in Table 4. The numerous parameters involved in the equations of the present model were estimated from available experimental data and mathematical models wherever possible. For example, *D*_*L*_ the diffusion coefficient of LDL was given by an in vitro experiment that calculated the average radioactivity with time, in the arteries incubated in Tyrode’s solution with radioactive tracer, and fitted the curve in which *D*_*L*_ as a parameter that can be given by the least-squares methods [25]. The coupled equations of all microenvironmental factors in this model are listed in S4 File. The nondimensionalization of the equations and the detailed parameter setting can be found in S5 and S6 Files and our previous work [16,17]. After nondimensionalization of all equations, the results of variants representing the plaque microenvironment are dimensionless and can be comparable among patients. To avoid the pre-defined interventions as much as possible, we assumed that the interaction coefficients between the microenvironmental factors remain unchanged during plaque progression. Coupled diffusion-reaction equations were solved by Euler finite difference method simultaneously (the numerical schemes to discretize the equations can be found in S7 File).

**Table 4:** The detailed explanations of reaction terms involved in the model system.

Variables	Reaction Terms	Explanation
LDL: ***L***	−***λ***_***L***_***L***	Lipid oxidation [9]
	$\lambda_{Pl_{extra}}Pl_{extra}$	LDL from leaky microvessels [2]
ox-LDL: ***L***_***ox***_	***λ***_***Lox⋅L***_***L***	ox-LDL formed by oxidation of LDL [19]
	$-\lambda_{L_{ox}\cdot Ma}L_{ox}Ma$	Macrophage phagocytosis of ox-LDL [19]
MCP-1: ***P***	$\lambda_{P\cdot E}\frac{L_{ox}}{K_{P}+L_{ox}}E$	Production of MCP-1 produced by ECs [17]
	***λ***_***P⋅S***_***S***	Production of MCP-1 by SMCs [26]
	**−*d***_***p***_***P***	Decay of MCP-1 [2,26]
Macrophage: ***Ma***	**−∇(λ**_**Ma⋅P**_***Ma*∇*P*)**	Chemotaxis of macrophages in response to MCP-1 [9]
	**λ**_**Ma⋅Mo**_***Mo***	Macrophages differentiated from monocytes [9]
	**−*d***_***Ma***_***Ma***	Apoptosis of macrophages [9]
Monocyte: **Mo**	$\nabla (\lambda_{Mo\cdot L_{ox}}Mo\nabla L_{ox})$	Chemotaxis of monocytes in response to ox-LDL [9]
	$\lambda_{Pl_{extra}}Pl_{extra}$	Monocytes from leaky microvessels [2]
	**−*d***_***Mo***_***Mo***	Decay of monocytes [19]
ECs: ***E***	$-\nabla (\frac{\lambda_{E\cdot C_{v}}}{K_{E}+C_{v}}E\nabla C_{v})$	Chemotaxis of ECs in response to VEGF [2]
	$\nabla (\lambda_{E\cdot C_{ECM}}E\nabla C_{ECM})$	Haptotaxis of ECs in response to the ECM [2]
VEGF: ***C***_***v***_	$-\lambda_{C_{v}\cdot E}E$	VEGF uptake by ECs [2]
	$\lambda_{C_{v}\cdot S}S$	VEGF early production by SMCs [2]
	$\lambda_{C_{v}\cdot Ma}Ma$	VEGF late production by Macrophages [2]
	$-d_{C_{v}}C_{v}$	Decay of VEGF [2]
Plasma: ***Pl***_***extra***_	**−*ψ*∇(*U***_***i***_***Pl***_***extra***_**)**	Convection of plasma [2]
	***γQ***_***t***_	Fluid flux of plasma [2]
SMCs: ***S***	**−∇(*λ***_***S*⋅*P***_***S*∇*P*)**	Chemotaxis of SMCs in response to MCP-1 [26]
	**−∇(*λ***_***S*⋅*Ma***_***S*∇*Ma*)**	Chemotaxis of SMCs in response to PDGF secreted from macrophages [26]
	$\nabla (\lambda_{S\cdot C_{ECM}}S\nabla C_{ECM})$	Haptotaxis of SMCs in response to ECM [26]
	**−*d***_***S***_***S⋅L***_***ox***_	Apoptosis of SMCs induced by ox-LDL [27,28]
ECM: ***C***_***ECM***_	$-\lambda_{C_{M}\cdot C_{ECM}}C_{ECM}C_{M}$	ECM degraded by MMPs [2]
	$\lambda_{S\cdot C_{ECM}}S$	ECM produced by SMCs [26]
MMP: ***C***_***M***_	$\lambda_{C_{M}\cdot E}E$	MMPs produced by ECs [2]
	$\lambda_{C_{M}\cdot S}S$	MMPs produced by SMCs [19]
	$-d_{C_{M}}C_{M}$	Decay of MMPs [2,19]

**Fig 7:**
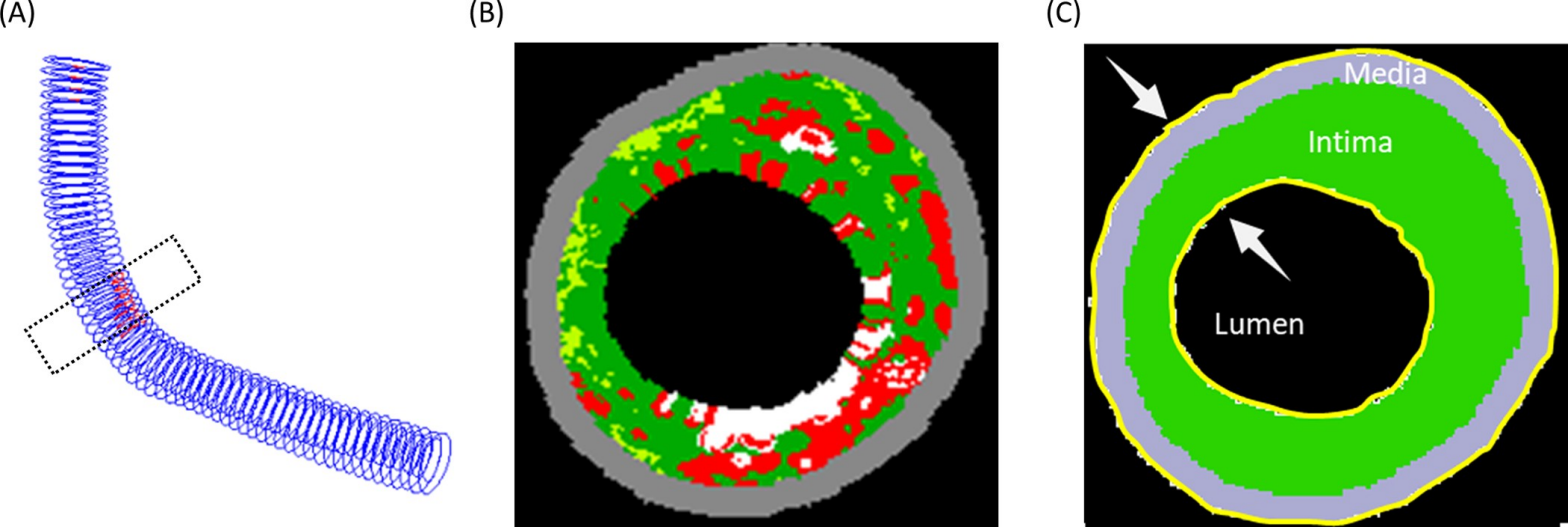
Patient-specific model generated from VH-IVUS image. A schematic for the geometry derived from patient-specific VH-IVUS data. (A) Three-dimensional vessel model reconstructed from IVUS series, and correlated between T1 and T2 slice by slice, which was performed in our previous work [29]. The dotted box shows the plaque area. (B) The slice with maximum plaque burden (PB) at T1. (C) The simulation region extracted from VH-IVUS image, where the region separated into three part: media (grey), thickening intima (green) and lumen (black), and the arrows pointed to the inner boundary (endothelium layer) and outer boundary (external elastic membrane) respectively.

The mathematical model was based on patient-specific VH-IVUS image (Fig 7), which was a 2D simulation domain of 4mm*4mm and divided uniformly into 200*200 grids, i.e., the space length for each grid was 20μm. Considering the limited resolution of IVUS and the co-registration of images at two-time points, the slice with the maximum plaque burden (PB) at T1 was chosen as initial condition to reflect the geometry of the patient’s plaque. Here, we used the definition of plaque burden as: *plaque burden* = *plaque area*/(*plaque area*+*lumen area*). Furthermore, the eccentricity index (EI) was considered as a morphology factor affected the risk of plaque, which was given by: EI = 1−*WT*_*Min*_*/WT*_*Max*_, where WT_Min_, WT_max_ were the minimum and maximum wall thickness in the plaque.

We primarily considered the morphological information provided by the VH-IVUS images and set up three regions accordingly: media, thickening intima, and lumen as shown in Fig 7. The outer boundary was set to be the external elastic membrane (EEM), while the inner one was the endothelium. The colored region in VH-IVUS images was defined as the thickened intima. The Dirichlet boundary condition was applied at the outer boundary where no substance exchange and cells migration happened. Also we assumed that the geometry of outer boundary had no changes during the simulation. The patient data at T1, including the plaque geometry, the plasma LDL, and the local WSS, were set as initial conditions of the simulation. The accumulation of LDL and monocytes into the plaque lesion lay on two distinct ways, one was from the abnormal angiogenic microvessels and the other was the transluminal transport from the injured endothelium. In this model, the former mechanism was assumed to be related with the extravascular plasma concentration, i.e., the intraplaque hemorrhage. The latter mechanism was controlled by the plasma LDL or monocyte level (*C*_*LDL*_/*C*_*Mo*_) and local WSS level, i.e., the influx of LDL and monocytes from the lumen into the intima. The solute flux per unit into endothelium of LDL and monocytes are denoted by J_LDL_ and J_Mo_, which were satisfied:

$$J_{LDL}=L_{cr}\frac{C_{LDL}}{1+\frac{WSS}{WSS_{0}}},$$ (2)

$$J_{Mo}={Mo}_{cr}\frac{C_{Mo}}{1+\frac{WSS}{WSS_{0}}},$$ (3)

where WSS and *C*_*LDL*_ were obtained from Table 3; *WSS*_0_ = 100 *dyn*·*cm*^−2^ [29–31] and the conductive rate L_cr_ for LDL, *Mo*_*cr*_ for monocytes, equaled to 2×10^−5^m·s^−1^, 4×10^−5^*m*·*s*−1, respectively [32]. *C*_*Mo*_ represented the concentration of monocyte in plasma, and was set to be 4×10^5^ *cells*·*cm*^−3^, 8×10^5^ *cells*·*cm*^−3^ and 16×10^5^ *cells*·*cm*^−3^, corresponding to mild, moderate and severe inflammatory levels respectively.

The SMCs were assumed to migrate from the media into the intima. Hence, an initial concentration of SMCs of 6×10^−3^*g*·*cm*^−3^ in media and 3×10^−3^*g*·*cm*^−3^ in intima was defined. The initial concentration of LDL and ox-LDL were set to be 1.0×10^−3^*mg*·*mm*^−2^ and 1×10^−7^*mg*·*mm*^−2^, and distributed in the intima evenly. MMP, MCP-1 and ECM were initialized to be 3×10^−8^*g*·*cm*^−3^, 3×10^−10^*g*·*cm*^−3^ and 4×10^−2^*g*·*cm*^−3^, respectively. The average concentration of VEGF was 4×10^−10^ *g*·*cm*^−3^, and a concentration gradient from media towards intima was set to stimulate neovascularization [16,17].

### Microenvironment analysis

We chose the changes of NC area and plaque burden (area) as the evaluation indicators of plaque development. In the simulation results, the plaque area indicated the area between the lumen and the outer boundary. The NC area was calculated by the apoptotic SMCs, i.e., the area where SMCs decreased more than 50% compared with their initial value. Since the initial NC area varies for each patient, we used the growth rate of NC to characterize plaque development, i.e. *growth rate* = *N*(*t*)/*N*_0_, where *N*(*t*) was the NC area in simulation result at the time point *t*, and *N*_0_ was the initial NC area at T1.

Since the dynamic distribution of the main factors in the microenvironment can be obtained from the simulation, a scoring scale was proposed to quantify the severity of the atherosclerosis, which comprised the morphological index (plaque burden and eccentric index, at T1), the lipid panel test (LDL and HDL, at T1), the local hemodynamic factor (WSS, at T1), and the representative intraplaque microenvironmental factors (macrophages, SMCs, and ox-LDL, at T3). There were three grades on the scale. A larger number indicated a more severe influence on plaque progression (1 score = mild; 2 score = moderate; 3 score = severe). Due to the individual variation of the influence of risk factors on the plaque development, no threshold of grading for each factor was predefined. The relative scales were determined by comparing the respective values of these four patients to obtain the relative value of the severity. Therefore, the present scoring system was only applicable to the patients in this study. The severity of microenvironmental factors was defined by the concentration of each variable at T3, while the influence of other risk factors was estimated according to the current consensus. For example, a higher LDL and a lower HDL concentration were demonstrated as blood indicators of a higher probability that a plaque may develop [8]. And a higher plaque burden and/or eccentric index, as the morphological factors, indicated that the plaque is more dangerous [33–35]. In terms of hemodynamics, it was found that low and/or oscillatory shear stress contributes to atherogenesis.

## Supporting information

S1 File Dynamic processes of microenvironmental factors. Files in.MPEG format presented the changes of eight factors are included in an archived ZIP file. Each file demonstrates the change in concentration and distribution of one factor, where time span is from T1 (baseline) to T3 (three years later). All values in the animate are normalized to range from zero to one.
(ZIP)

S2 File Identification of initial inflammation and neovascularization. (DOCX)

S3 File The fluid-structure interaction (FSI) model. (DOCX)

S4 File The coupled equations of plaque progression model. (DOCX)

S5 File Nondimensionalization of the partial differential equations. (DOCX)

S6 File The setting of the parameters in the model. (DOCX)

S7 File The scheme of finite differential method. (DOCX)
