# Correction: A prediction tool for plaque progression based on patient-specific multi-physical modeling

**DOI:** 10.1371/journal.pcbi.1009709

**Published:** 2021-12-28

**Authors:** Jichao Pan, Yan Cai, Liang Wang, Akiko Maehara, Gary S. Mintz, Dalin Tang, Zhiyong Li

There is an error in Paragraph 1 of “Image-based patient-specific multi-physical modelling” in the Methods section. The correct sentence should read:

The reaction terms of *C*_*i*_ were modeled based on the proved pathophysiological knowledge during the plaque progression, including the production, consumption, chemotaxis, haptotaxis, and differentiation by other microenvironmental factor *C*_*j*_, as well as the apoptosis by itself.
