# Supplementary material for: A prediction tool for plaque progression based on patient-specific multi-physical modeling
Source: PLoS Comput Biol. 2021 Mar 29;17(3):e1008344. doi: 10.1371/journal.pcbi.1008344 (PMC8057612; doi:10.1371/journal.pcbi.1008344)
Supplement: S2 File — (DOCX) [file pcbi.1008344.s002.docx]

**S2. Identification of initial inflammation and neovascularization**

Since the initial condition of inflammation and microvessels inside the plaque cannot be provided by VH-IVUS data, multiple simulations for each patient are carried out to obtain the plaque development under three different levels of microenvironment, namely low, normal and high levels. For inflammation, it was defined as different initial macrophage and monocyte concentration within intima and monocyte flux into lumen boundary; for angiogenesis, it was based on different sprout numbers.

By comparing the simulated NC growth with changes of NC area based on VH-IVUS during the time interval, the inflammatory microenvironment and neovascularization at T1 can be identified correspondingly (Table S2-1). It is noteworthy that the density of neovasculature is assumed consistency with the degree of inflammation. The initial inputs for the dynamic modeling of given patient comprise the identified microenvironment (inflammation and neovascularization) as well as the patient-derived data (plaque composition, plasma LDL, and local WSS).

Table S2-1. The growth rates of necrotic core of four patients

| No. | Low | Normal | High | IVUS |
| --- | --- | --- | --- | --- |
| P1 | 0.4390* | 0.9611 | 1.1424 | 0.4491 |
| P2 | 0.3389* | 0.4465 | 1.1344 | 0.2587 |
| P3 | 1.2224 | 0.3577 | 0.4066* | 0.4938 |
| P4 | 1.2166 | 1.4586 | 1.8453* | 1.8283 |

* indicates the plaque progression with certain initial microenvironment that is most similar to the realistic NC development observed by VH-IVUS.
