# Supplementary material for: A prediction tool for plaque progression based on patient-specific multi-physical modeling
Source: PLoS Comput Biol. 2021 Mar 29;17(3):e1008344. doi: 10.1371/journal.pcbi.1008344 (PMC8057612; doi:10.1371/journal.pcbi.1008344)
Supplement: S3 File — (DOCX) [file pcbi.1008344.s003.docx]

S3. The fluid-structure interaction (FSI) model:

1. *The FSI model and boundary conditions*

The IVUS imaging based 3-D FSI model with cyclic bending was developed by Wang et al (1,2). In this model, blood flow was assumed to be laminar, Newtonian, and incompressible. Cyclic bending was specified by prescribing periodic displacement at the myocardium side of the vessel using data obtained from X-Ray angiography. The vessel material was assumed to be hyperelastic, anisotropic, nearly-incompressible and homogeneous. Plaque components were assumed to be hyperelastic, isotropic, nearly-incompressible and homogeneous for simplicity. The vessel material data was obtained by biaxial testing using eight coronary arteries from 4 cadavers. A modified Mooney-Rivlin model was used for the vessel fitting the biaxial data:
$W=c_{1}\left( I_{1}-3 \right)+c_{2}\left( I_{2}-3 \right)+D_{1}\left[ \exp\left( D_{2}\left( I_{1}-3 \right) \right)-1 \right]+K_{1}/2K_{2}\{\exp\left[ K_{2}\left( I_{4}-1 \right)^{2}-1 \right]\}$.
$I_{1}=\sum C_{\mathrm{ii}}, I_{2}=1/2[I_{1}^{2}-C_{ij}C_{ij}]$,
where $I_{1}$ and $I_{2}$ are the first and second invariants of right Cauchy-Green deformation tensor $\mathbf{C}$ defined as $\mathbf{C}=\left[ C_{\mathrm{ij}} \right]=\boldsymbol{X}^{T}\boldsymbol{X, X=}\left[ X_{ij} \right]=[\partial x_{i}/\partial a_{j}]$, ($x_{i}$) is current position, ($a_{j}$) is original position, $I_{4}=C_{ij}\left( \boldsymbol{n}_{c} \right)_{i}\left( \boldsymbol{n}_{c} \right)_{j}$, $\boldsymbol{n}_{c}$ is the unic vector in the circumferential direction of the vessel, $c_{1}, D_{1}, D_{2}$ and $K_{1}$ and $K_{2}$ are material constants. The parameter values used in this model were:$c_{1}=-1312.9 \mathrm{kPa}$, $c_{2}=114.7 \mathrm{kPa}$, $D_{1}=629.7 \mathrm{kPa}$, $D_{2}=2.0$, $K_{1}=35.9 \mathrm{kPa}$, $c_{1}=23.5$.

1. *Plaque geometry and mesh generation*

The 3D coronary plaque FSI models for all the patients were reconstructed and solved by ADINA (Adina R & D, Watertown, MA). The 3D vessel domain was divided into hundreds of small ‘‘volumes’’ to fit the irregular vessel geometry with plaque components inclusions. The element type used for structural models (vessel and plaque components) was 3D solid 8-node element while the element type used for the fluid model was 3D 4-node element, free formed mesh. More details of the computational models and solution methods can be found in Tang et al (3).

**Reference:**

1. Wang L, Zheng J, Maehara A, Yang C, Billiar KL, Wu Z, et al. Morphological and Stress Vulnerability Indices for Human Coronary Plaques and Their Correlations with Cap Thickness and Lipid Percent: An IVUS-Based Fluid-Structure Interaction Multi-patient Study. McCulloch AD, editor. PLoS Comput Biol. 2015 Dec 9;11(12):e1004652.

2. Wang L, Wu Z, Yang C, Zheng J, Bach R, Muccigrosso D, et al. IVUS-Based FSI Models for Human Coronary Plaque Progression Study: Components, Correlation and Predictive Analysis. Ann Biomed Eng. 2015 Jan;43(1):107–21.

3. Tang D, Kamm RD, Yang C, Zheng J, Canton G, Bach R, et al. Image-based modeling for better understanding and assessment of atherosclerotic plaque progression and vulnerability: Data, modeling, validation, uncertainty and predictions. Journal of Biomechanics. 2014 Mar 3;47(4):834–46.
