# Supplementary material for: A prediction tool for plaque progression based on patient-specific multi-physical modeling
Source: PLoS Comput Biol. 2021 Mar 29;17(3):e1008344. doi: 10.1371/journal.pcbi.1008344 (PMC8057612; doi:10.1371/journal.pcbi.1008344)
Supplement: S4 File — (DOCX) [file pcbi.1008344.s004.docx]

**S4. The coupled equations of plaque progression model**

We model the cellular and acellular components involved in four main pathophysiological processes during plaque development, i.e., lipid deposition, inflammatory response, migration and proliferation of SMCs, and neovascularization. In particular, the cellular components consist of endothelial cells (ECs), macrophages (MΦs), monocytes (MOs), foam cells (FCs) and SMCs, while the acellular counterparts include LDL, ox-LDL, MCP-1, VEGF, MMP, ECM and extravascular plasma concentration. The dynamics of these twelve variations in this model satisfy the mass conservation, and we describe them with a system of coupled reaction-diffusion equations, Eq.(S4-1)-(S4-14). The coupled changes of microenvironmental factors are described by the reaction terms in the equations. For example, Eq. (S4-2) models the production of ox-LDL due to LDL oxidation by reaction with the radicals (second term on right-hand side) and the reduction of ox-LDL through ingestion by macrophages (third term on right-hand side).

| Variable | symbol | Variable | symbol | Variable | symbol |
| --- | --- | --- | --- | --- | --- |
| LDL | L | Macrophage | Ma | Leakage plasma | $Pl_{extra}$ |
| ox-LDL | $L_{ox}$ | Monocyte | Mo | SMCs | S |
| Foam cell | F | ECs | E | ECM | $C_{ECM}$ |
| MCP-1 | P | VEGF | $C_{v}$ | MMP | $C_{M}$ |

$\frac{\partial L}{\partial t}=D_{L}\nabla^{2}L-\lambda_{L}L$ (S4-1)

$\frac{\partial L_{ox}}{\partial t}=D_{L_{ox}}\nabla^{2}L_{ox}+\lambda_{L_{ox}\cdot L}L-\lambda_{L_{ox}\cdot Ma}L_{ox}Ma$ (S4-2)

$\frac{\partial F}{\partial t}=\lambda_{L_{ox}\cdot Ma}L_{ox}Ma$ (S4-3)

$\frac{\partial P}{\partial t}=D_{P}\nabla^{2}P-\lambda_{P\cdot E}\left( \frac{L_{ox}}{K_{P}+L_{ox}}E \right)+\lambda_{P\cdot S}S-d_{p}D$ (S4-4)

$\frac{\partial Ma}{\partial t}=D_{Ma}\nabla^{2}Ma-\nabla\left( \mu_{Ma}\cdot Ma \right)-\nabla\left( \lambda_{Ma\cdot P}Ma\nabla P \right)+\lambda_{Ma\cdot Mo}Mo-d_{Ma}Ma$ (S4-5)

$\frac{\partial Mo}{\partial t}=D_{Mo}\nabla^{2}Mo-\nabla\left( \lambda_{Mo\cdot L_{ox}}Mo\nabla L_{ox} \right)-d_{Mo}Mo$ (S4-6)

$\frac{\partial E}{\partial t}=D_{E}\nabla^{2}E-\nabla\left( \frac{\lambda_{E\cdot C_{v}}}{K_{E}+C_{v}}E\nabla C_{v} \right)-\nabla(\lambda_{E\cdot C_{ECM}}E\nabla C_{ECM})$ (S4-7)

$\frac{\partial C_{v}}{\partial t}=D_{C_{v}}\nabla^{2}C_{v}-\lambda_{C_{v}\cdot E}E+\lambda_{C_{v}\cdot S}S+\lambda_{C_{v}\cdot Ma}Ma-d_{C_{v}}C_{v}$ (S4-8)

$\frac{\partial Pl_{extra}}{\partial t}=\varphi\nabla^{2}Pl_{extra}-\psi\nabla\left( U_{i}Pl_{extra} \right)+\gamma Q_{t}$ (S4-9)

$\frac{\partial L}{\partial t}=D_{L}\nabla^{2}L-\lambda_{L}L+\lambda_{Pl_{extra}}Pl_{extra}$ (S4-10)

$\frac{\partial Mo}{\partial t}=D_{Mo}\nabla^{2}Mo-\nabla\left( \lambda_{Mo\cdot L_{ox}}Mo\nabla L_{ox} \right)-d_{Mo}Mo+\lambda_{Pl_{extra}}Pl_{extra}$ (S4-11)

$\frac{\partial S}{\partial t}=D_{S}\nabla^{2}S-\nabla\left( \lambda_{S\cdot P}S\nabla P \right)-\nabla\left( \lambda_{S\cdot Ma}S\nabla Ma \right)-\nabla\left( \lambda_{S\cdot C_{ECM}}S\nabla C_{ECM} \right)-d_{S}L_{ox}S$ (S4-12)

$\frac{\partial C_{ECM}}{\partial t}=-\lambda_{C_{M}\cdot C_{ECM}}C_{ECM}C_{M}+\lambda_{S\cdot C_{ECM}}S$ (S4-13)

$\frac{\partial C_{M}}{\partial t}=D_{C_{M}}\nabla^{2}C_{M}+\lambda_{C_{M}\cdot E}E+\lambda_{C_{M}\cdot S}S-d_{C_{M}}C_{M}$ (S4-14)
