# Supplementary material for: A prediction tool for plaque progression based on patient-specific multi-physical modeling
Source: PLoS Comput Biol. 2021 Mar 29;17(3):e1008344. doi: 10.1371/journal.pcbi.1008344 (PMC8057612; doi:10.1371/journal.pcbi.1008344)
Supplement: S5 File — (DOCX) [file pcbi.1008344.s005.docx]

S5. Nondimensionalization of the partial differential equations

We nondimensionalize Eqs. (S4-1)-(S4-8) and Eqs. (S4-12)-(S4-14) by rescaling the distance with the diameter of the coronary artery (the thin adventitia is ignored), time with $\tau=l^{2}/D_{C_{v}}$ (where $D_{C_{v}}$ is VEGF diffusion coefficient), LDL density with $L_{0}$, ox-LDL density with $L_{ox0}$, MCP1 density with $P_{0}$, macrophages density with $Ma_{0}$, monocytes density with $Mo_{0}$, endothelial cell density with $E_{0}$, smooth muscle cells density with $S_{0}$, and VEGF, extracellular matrix concentration and matrix metalloproteinase with $C_{V0}$, $C_{ECM0}$, $C_{M0}$ respectively. Wherever possible the parameter values have been estimated from available experimental data. An average diameter of the coronary artery is between 2 and 4 mm, and we take the length scale $l=4 mm$. The diffusion coefficient of VEGF $D_{C_{V}}$ is in the range $0.5\times{10}^{-7}\sim2.8\times{10}^{-6} cm^{2}\cdot s^{-1}$ and we take $D_{C_{V}}=1\times{10}^{-7}cm^{2}\cdot s^{-1}$. $L_{0}$ is estimated to be $1.0\times{10}^{-3}g\cdot cm^{-3}$ for its range is $70-190 mg/dl$, $P_{0}$ is $3\times{10}^{-10} g\cdot cm^{-3}$ and $Mo_{0}$ is $4\times{10}^{-5}g\cdot cm^{-3}$ (range $2\times{10}^{5}\sim10\times{10}^{5} cells\cdot ml^{-1}$), which were calculated in Wenrui Hao et al (2014) (1). $L_{\mathrm{ox}0}$ is estimated to be ${1.2\times10}^{-6}g\cdot cm^{-3}$ due to the small amounts at the beginning. $E_{0}$ is estimated to be $8\times{10}^{-3}g\cdot{cm}^{-3}$. The research showed that concentration of VEGF ranges from $200 pg\cdot ml^{-1}$ to $400 pg\cdot ml^{-1}$, and we therefore take $C_{V0}=4\times{10}^{-10}g\cdot cm^{-3}$.

We set

$$\tilde{L}=\frac{L}{L_{0}}, \tilde{L_{ox}}=\frac{L_{ox}}{L_{ox0}},\tilde{F}=\frac{F}{F_{0}} \tilde{P}=\frac{P}{P_{0}}, \tilde{Ma}=\frac{Ma}{Ma_{0}}, \tilde{Mo}=\frac{Mo}{Mo_{0}}, \tilde{E}=\frac{E}{E_{0}}, \tilde{C_{V}}=\frac{C_{V}}{C_{V0}},$$

$$\tilde{S}=\frac{S}{S_{0}}, \tilde{C}_{ECM}=\frac{C_{ECM}}{C_{ECM0}}, \tilde{C}_{M}=\frac{C_{M}}{C_{M0}}, \tilde{t}=\frac{t}{\tau}$$

The process for nondimensionalizing Eq. (S4-1) is:

$$\frac{\partial L}{\partial t}=D_{L}\nabla^{2}L-\lambda_{L}L$$

$$\Rightarrow\frac{\partial\tilde{L}L_{0}}{\partial\tilde{t}\tau}=\frac{D_{L}\nabla^{2}\tilde{L}L_{0}}{l^{2}}-\lambda_{L}\tilde{L}L_{0}$$

$$\Rightarrow\frac{\partial\tilde{L}}{\partial\tilde{t}}=\frac{\tau D_{L}\nabla^{2}\tilde{L}}{l^{2}}-\tau\lambda_{L}\tilde{L}$$

and when $\tau=l^{2}/D_{C_{V}}$, we obtain the nondimensional equation:

$$\frac{\partial\tilde{L}}{\partial\tilde{t}}=\tilde{D}_{L}\nabla^{2}\tilde{L}-\tilde{\lambda}_{L}\tilde{L}$$

where
$\tilde{D}_{L}=\frac{D_{L}}{D_{C_{V}}}, \tilde{\lambda}_{L}=\frac{l^{2}\lambda_{L}}{D_{C_{V}}}$.
Nondimensionalization of Eqs. (S4-2)-(S4-8) and Eqs. (S4-12)-(S4-14) are similar to Eq.(S4-1)’s and finally we get,

$$\begin{matrix} & \tilde{D}_{L_{ox}}=\frac{D_{L_{ox}}}{D_{C_{V}}},\tilde{\lambda}_{L_{ox}\cdot L}=\frac{l^{2}\lambda_{L_{ox}L}L_{0}}{D_{C_{V}}L_{ox0}}, \tilde{\lambda}_{L_{ox}\cdot Ma}=\frac{l^{2}\lambda_{L_{ox}Ma}Ma_{0}}{D_{C_{V}}} \\ & \tilde{D}_{P}=\frac{D_{P}}{D_{C_{V}}},\tilde{\lambda}_{P\cdot E}=\frac{l^{2}\lambda_{P\cdot E}E_{0}}{D_{C_{V}}P_{0}},\beta=\frac{k_{P}}{L_{ox0}},\tilde{\lambda}_{P\cdot S}=\frac{l^{2}\lambda_{P\cdot S}S_{0}}{D_{C_{V}}P_{0}},\tilde{d}_{P}=\frac{l^{2}d_{P}}{D_{C_{V}}} \\ & \tilde{D}_{Ma}=\frac{D_{Ma}}{D_{C_{V}}},u_{Ma}=\frac{lu_{Ma}}{D_{C_{V}}},\tilde{\lambda}_{Ma\cdot P}=\frac{\lambda_{Ma\cdot P}P_{0}}{D_{C_{V}}},\tilde{\lambda}_{Ma\cdot Mo}=\frac{l^{2}\lambda_{Ma\cdot Mo}M_{o0}}{{Ma}_{0}D_{C_{V}}},\tilde{d}_{Ma}=\frac{l^{2}d_{Ma}}{D_{C_{V}}} \end{matrix}$$

$\begin{matrix} & \tilde{D}_{Mo}=\frac{D_{Mo}}{D_{C_{V}}},\tilde{\lambda}_{Mo\cdot L_{ox}}=\frac{\lambda_{Mo\cdot L_{ox}}L_{ox0}}{D_{C_{V}}},\tilde{d}_{Mo}=\frac{l^{2}d_{Mo}}{D_{C_{V}}} \\ & \tilde{D}_{E}=\frac{D_{E}}{D_{C_{V}}},\tilde{\lambda}_{E\cdot C_{V}}=\frac{C_{V0}\lambda_{E\cdot C_{V}}}{D_{C_{V}}},\alpha=\frac{C_{V0}}{k_{E}},\tilde{\lambda}_{E\cdot C_{ECM}}=\frac{C_{ECM0}\lambda_{E\cdot C_{BCM}}}{D_{C_{V}}} \\ & \tilde{D}_{S}=\frac{D_{S}}{D_{C_{V}}},\tilde{u}_{S}=\frac{lu_{S}}{D_{C_{V}}},\tilde{\lambda}_{S\cdot P}=\frac{\lambda_{S\cdot P}P_{0}}{D_{C_{V}}},\tilde{\lambda}_{S\cdot Ma}=\frac{\lambda_{S\cdot Ma}Ma_{0}}{D_{C_{V}}},\tilde{\lambda}_{S\cdot C_{ECM}}=\frac{\lambda_{S\cdot C_{ECM}}C_{ECM0}}{D_{C_{V}}} \\ & \tilde{\lambda}_{C_{ECM}\cdot C_{M}}=\frac{l^{2}\lambda_{C_{BCM}\cdot C_{M}}C_{M0}}{D_{C_{V}}},\tilde{\lambda}_{C_{ECM}\cdot S}=\frac{l^{2}\lambda_{C_{ECM}\cdot S}}{D_{C_{V}}} \\ & \tilde{D}_{C_{M}}=\frac{D_{C_{M}}}{D_{C_{r}}},\tilde{\lambda}_{C_{M}\cdot E}=\frac{l^{2}\lambda_{C_{M}\cdot E}E_{0}}{D_{C_{V}}C_{M0}},\tilde{\lambda}_{C_{M}\cdot S}=\frac{l^{2}\lambda_{C_{M}\cdot S}S_{0}}{D_{C_{V}}C_{M0}},\tilde{d}_{C_{M}}=\frac{l^{2}d_{C_{M}}}{D_{C_{V}}} \end{matrix}$

Table S5-1. The reference value

| Variable | Value | Variable | Value |
| --- | --- | --- | --- |
| $L_{0}$ | $1\times{10}^{-3}g\cdot cm^{-3}$ | $P_{0}$ | $3\times{10}^{-7}g\cdot cm^{-3}$ |
| ${L_{\mathrm{ox}}}_{0}$ | $1\times{10}^{-4}g\cdot cm^{-3}$ | $S_{0}$ | $6\times{10}^{-3}g\cdot cm^{-3}$ |
| $\mathrm{Mo}_{0}$ | $4\times{10}^{5}cells\cdot cm^{-3}$ | ${C_{E}}_{0}$ | $8\times{10}^{-3}g\cdot cm^{-3}$ |
| $\mathrm{Ma}_{0}$ | $4\times{10}^{5}cells\cdot cm^{-3}$ | $F_{0}$ | $4\times{10}^{4}cells\cdot cm^{-3}$ |
| $C_{v}$ | $4\times{10}^{-10}g\cdot cm^{-3}$ | $C_{\mathrm{ECM}_{0}}$ | $4\times{10}^{-2}g\cdot cm^{-3}$ |
| $C_{M0}$ | $3\times{10}^{-8}g\cdot cm^{-3}$ |  |  |

All the reference values are from our previous work (2,3).

**Reference**

1. Hao W, Friedman A. The LDL-HDL Profile Determines the Risk of Atherosclerosis: A Mathematical Model. PLoS One. 2014 Mar 12;9(3):e90497.

2. Guo M, Cai Y, Yao X, Li Z. Mathematical modeling of atherosclerotic plaque destabilization: Role of neovascularization and intraplaque hemorrhage. J Theor Biol. 2018 Aug;450:53–65.

3. Guo M, Cai Y, He C, Li Z. Coupled Modeling of Lipid Deposition, Inflammatory Response and Intraplaque Angiogenesis in Atherosclerotic Plaque. Ann Biomed Eng. 2019 Feb;47(2):439–52.
