# Supplementary material for: A prediction tool for plaque progression based on patient-specific multi-physical modeling
Source: PLoS Comput Biol. 2021 Mar 29;17(3):e1008344. doi: 10.1371/journal.pcbi.1008344 (PMC8057612; doi:10.1371/journal.pcbi.1008344)
Supplement: S6 File — (DOCX) [file pcbi.1008344.s006.docx]

S6. The setting of the parameters in the model:

Description and value of the parameters

| **Parameter** | **Description** | **Value** |
| --- | --- | --- |
| $L_{0}$ | LDL density | $7\times{10}^{-4}\sim1.9\times{10}^{-3}g/cm^{3}$ (1) ($1.0\times{10}^{-3}g/cm^{3}$)* |
| $L_{ox0}$ | ox-LDL density | $10\times{10}^{-8}g/cm^{3}$ estimated |
| $P_{0}$ | MCP-1 density | $3\times{10}^{-10}g/cm^{3}$ (1) |
| $Ma_{0}$ | Macrophages density | $4\times{10}^{-5}g/cm^{3}$ estimated |
| $Mo_{0}$ | Monocytes density | $2\times{10}^{4}\sim2\times{10}^{5}$cells per ml (1) |
| $E_{0}$ | Endothelial cell density | $8\times{10}^{-3}g/cm^{3}$ estimated |
| $C_{v0}$ | VEGF concentration | $200\sim400 pg/ml$ (2)  ($400 pg/ml)$* |
| $S_{0}$ | Smooth muscle cells density | $7.5\times{10}^{6}-{10}^{7}$cells per ml (1)  ($6\times{10}^{-3}g/cm^{3}$)* |
| $C_{M0}$ | Matrix metalloproteinase | $10-60 ng/ml$ (1) ($30 ng/ml$)* |
| $C_{\mathrm{EMC}0}$ | Extracellular matrix concentration | $4\times{10}^{-2}g/cm^{3}$ (3) & estimated |
| $D_{L}$ | Diffusion coefficient of LDL | $8\times{10}^{-8}cm^{2}/s$ (4) & estimated |
| $D_{L_{ox}}$ | Diffusion coefficient of ox-LDL | $8\times{10}^{-8}cm^{2}/s$ (4) & estimated |
| $D_{P}$ | Diffusion coefficient of MCP-1 | $2\times{10}^{-4}cm^{2}/s$ (1) |
| $D_{Ma}$ | Diffusion coefficient of macrophages | $1\times{10}^{-11}cm^{2}/s$ (1) |
| $D_{Mo}$ | Diffusion coefficient of monocytes | $1\times{10}^{-5}cm^{2}/s$ (4) |
| $D_{E}$ | Diffusion coefficient of ECs | $1\times{10}^{-10}cm^{2}/s$ (5,6) |
| $D_{C_{v}}$ | Diffusion coefficient of VEGF | $1\times{10}^{-7}cm^{2}/s$ (5) |
| $D_{S}$ | Diffusion coefficient of SMCs | $1\times{10}^{-11}cm^{2}/s$ (5) |
| $D_{C_{M}}$ | Diffusion coefficient of MMP | $5\times{10}^{-12}cm^{2}/s$ (5) |
| $\lambda_{L}$ | Reduction rate of LDL | $3\times{10}^{-4}/s$ (4) |
| $\lambda_{L_{ox}\cdot L}$ | Rate of ox-LDL oxidized from LDL | $2.7\times{10}^{-9}/s$ (1) & estimated |
| $\lambda_{L_{ox}\cdot Ma}$ | Rate of ox-LDL ingestion by macrophages | $1.16\times{10}^{-4}g/cm^{3}\cdot s$ (1) |
| $\lambda_{P\cdot E}$ | Rate of the production of MCP-1 by ECs | $1\times{10}^{-14}/s$ (1) |
| $\lambda_{P\cdot S}$ | Rate of the production of MCP-1 by SMCs | $5.8\times{10}^{-6}/s$ (5) & estimated |
| $\lambda_{Ma\cdot P}$ | Chemotactic sensitivity parameter about Macrophages and MCP-1 | $5\times{10}^{-9}cm^{5}/g\cdot s$ (5) |
| $\lambda_{Ma\cdot Mo}$ | Differentiation rate of monocytes to macrophages | $2.7\times{10}^{-9}/s$ (6) |
| $\lambda_{Mo\cdot L_{ox}}$ | Chemotactic sensitivity parameter about monocytes and ox-LDL | $1\times{10}^{-8}cm^{5}/g\cdot s$ (5) & estimated |
| $\lambda_{E\cdot C_{v}}$ | Chemotactic sensitivity parameter about ECs and VEGF | $1\times{10}^{-9}cm^{5}/g\cdot s$ (5) |
| $\lambda_{E\cdot C_{ECM}}$ | Haptotaxis parameter about ECs and ECM | $7\times{10}^{-10}cm^{5}/g\cdot s$ (5) |
| $\lambda_{C_{v}\cdot E}$ | Uptake rate of VEGF by ECs | $7.5\times{10}^{-6}/s$ (5) |
| $\lambda_{C_{v}\cdot S}$ | Rate of the production of VEGF by SMCs | $5.8\times{10}^{-6}/s$ (5) |
| $\lambda_{C_{v}\cdot Ma}$ | Rate of the production of VEGF by macrophages | $5.8\times{10}^{-6}/s$ (5) & estimated |
| $\lambda_{S\cdot P}$ | Chemotactic sensitivity parameter about SMCs and MCP-1 | $1\times{10}^{-8}cm^{5}/g\cdot s$ (5) & estimated |
| $\lambda_{S\cdot Ma}$ | Chemotactic sensitivity parameter about SMCs and macrophages | $1\times{10}^{-8}cm^{5}/g\cdot s$ (5) |
| $\lambda_{S\cdot C_{ECM}}$ | Haptotaxis parameter about SMCs and ECM | $5\times{10}^{-10}cm^{5}/g\cdot s$ (5) |
| $\lambda_{C_{ECM}\cdot C_{M}}$ | Degradation rate of ECM by MMP | $2.5\times{10}^{-6}cm^{3}/g\cdot s$ (5) |
| $\lambda_{C_{ECM}\cdot S}$ | Rate of the production of ECM by SMCs | $5.8\times{10}^{-6}/s$ (5) & estimated |
| $\lambda_{C_{M}\cdot E}$ | Rate of the production of MMP by ECs | $3.47\times{10}^{-9}/s$ (5) |
| $\lambda_{C_{M}\cdot S}$ | Rate of the production of MMP by SMCs | $3.47\times{10}^{-9}/s$ (5) |
| $\lambda_{Pl_{extra}}$ | Supplement of LDL or monocyte from IPH | $5\times{10}^{-9}/s$ (6) & estimated |
| $d_{P}$ | Decay rate of MCP-1 | $2\times{10}^{-5}/s$ (1) |
| $d_{Ma}$ | Death rate of macrophages | $1.74\times{10}^{-7}/s$ (1) |
| $d_{Mo}$ | Decay rate of monocytes | $1.74\times{10}^{-7}/s$ (1) |
| $d_{C_{v}}$ | Decay rate of VEGF | $1\times{10}^{-5}/s$ (5) |
| $d_{C_{M}}$ | Decay rate of MMP | $5\times{10}^{-10}/s$ (5) |
| $k_{P}$ | ox-LDL saturation for production of MCP-1 | $5\times{10}^{-1}g/cm^{3}$ (1) |
| $k_{E}$ | VEGF saturation for production of ECs | $3.5\times{10}^{-10}g/cm^{3}$ (5) |
| $\varphi$ | Diffusion coefficient of IPH | $0.4$(5) |
| $\psi$ | Influence of the convection | $0.05$ (5) |
| $\gamma$ | Influence of fluid | $0.4$ (5) |

* Values chosen in the simulation.

**Reference**

1. Hao W, Friedman A. The LDL-HDL Profile Determines the Risk of Atherosclerosis: A Mathematical Model. PLoS One. 2014 Mar 12;9(3):e90497.

2. Obermair A, Tempfer C, Hefler L, Preyer O, Kaider A, Zeillinger R, et al. Concentration of vascular endothelial growth factor (VEGF) in the serum of patients with suspected ovarian cancer. British Journal of Cancer. 1998 Jun;77(11):1870–4.

3. Ghuman H, Massensini AR, Donnelly J, Kim S-M, Medberry CJ, Badylak SF, et al. ECM hydrogel for the treatment of stroke: Characterization of the host cell infiltrate. Biomaterials. 2016 Jun 1;91:166–81.

4. Guilia DT, Díaz-Zuccarini V, Pichardo-Almarza C. A Multiscale Model of Atherosclerotic Plaque Formation at Its Early Stage. IEEE Trans Biomed Eng. 2011 Dec;58(12):3460–3.

5. Guo M, Cai Y, Yao X, Li Z. Mathematical modeling of atherosclerotic plaque destabilization: Role of neovascularization and intraplaque hemorrhage. J Theor Biol. 2018 Aug;450:53–65.

6. Guo M, Cai Y, He C, Li Z. Coupled Modeling of Lipid Deposition, Inflammatory Response and Intraplaque Angiogenesis in Atherosclerotic Plaque. Ann Biomed Eng. 2019 Feb;47(2):439–52.
