# Supplementary material for: A prediction tool for plaque progression based on patient-specific multi-physical modeling
Source: PLoS Comput Biol. 2021 Mar 29;17(3):e1008344. doi: 10.1371/journal.pcbi.1008344 (PMC8057612; doi:10.1371/journal.pcbi.1008344)
Supplement: S7 File — (DOCX) [file pcbi.1008344.s007.docx]

S7. The scheme of finite differential methods:

1. *The FTCS scheme*

For the general form of equations (Eq.1), it has four typical terms: the time derivation at left-hand sides, and diffusion, reaction, and production term at the right-hand sides. Hence, we considered the following form:

$$\frac{\partial u}{\partial t}=D_{u}\nabla^{2}u-\nabla u\left( \lambda\nabla v \right)+du$$

where $D_{u}$, $\lambda$ and $d$ are diffusion coefficient, reaction rate and production rate, respectively.

We divided a 2-D temporal-spatial region, $\left[ 0,N \right]\times[0,M]\times[0,T_{F}]$, into $n\times m\times T$ elements and wrote $u_{i,j}^{t}=\left( i\Delta x, j\Delta y, t\Delta t \right), i=0, \ldots,n, j=0,\ldots, m, t=0, \ldots, T$. We used forward time difference and central space difference and obtained the scheme:

$$\frac{u_{i,j}^{t+1}-u_{i, j}^{t}}{\Delta t}= \frac{D_{u}\left( u_{i+1, j}^{t}-{2u}_{i, j}^{t}+u_{i-1, j}^{t}+u_{i, +1j}^{t}-{2u}_{i, j}^{t}+u_{i, j-1}^{t} \right)}{\left( \Delta x \right)^{2}}$$

$$- \lambda\frac{\left( u_{i+1, j}^{t}-u_{i-1, j}^{t} \right)}{2\Delta x}\times\frac{\left( v_{i+1, j}^{t}-v_{i-1, j}^{t} \right)}{2\Delta x}$$

$$- \lambda\frac{\left( u_{i, j+1}^{t}-u_{i, j-1}^{t} \right)}{2\Delta x}\times\frac{\left( v_{i, j+1}^{t}-v_{i, j-1}^{t} \right)}{2\Delta x}$$

$$- \lambda u_{i,j}\frac{\left( v_{i+1, j}^{t}-{2v}_{i, j}^{t}+v_{i+1, j}^{t}+v_{i, j+1}^{t}-{2v}_{i, j}^{t}+v_{i, j-1}^{t} \right)}{\left( \Delta x \right)^{2}}$$

$$+ du_{i, j}^{t}$$

1. *Discretization of equation*

We rewrote Eq.1 as:

$\frac{\partial u}{\partial t}=D_{u}\nabla^{2}u+\lambda_{P}v-\lambda_{C}v-\lambda_{A}u-\nabla\left( \lambda_{TC}u\nabla v \right)-\nabla\left( \lambda_{Th}u\nabla v \right)-\lambda_{de}uv$ (S7-1)

where $u,v$ representing $C_{i}, C_{j}$ in Eq.1, respectively.

We used FTCS method to discretize Eq.(S7-1):

$$\frac{u_{i,j}^{t+1}-u_{i,j}^{t}}{\Delta t}=D_{u}\frac{\left( u_{i+1,j}^{t}+u_{i-1,j}^{t}+u_{i,j+1}^{t}+u_{i,j-1}^{t}-4u_{i,j}^{t} \right)}{\left( \Delta x \right)^{2}}+\lambda_{P}v_{i,j}^{t}-\lambda_{C}v_{i,j}^{t}-\lambda_{A}u_{i,j}^{t}$$

$$-\lambda_{TC}\left[ \frac{\left( u_{i+1, j}^{t}-u_{i-1, j}^{t} \right)}{2\Delta x}\times\frac{\left( v_{i+1, j}^{t}-v_{i-1, j}^{t} \right)}{2\Delta x}+\frac{\left( u_{i, j+1}^{t}-u_{i, j-1}^{t} \right)}{2\Delta x}\times\frac{\left( v_{i, j+1}^{t}-v_{i, j-1}^{t} \right)}{2\Delta x}+u_{i,j}^{t}\frac{\left( v_{i+1, j}^{t}+v_{i+1, j}^{t}+v_{i, j+1}^{t}+v_{i, j-1}^{t}-{4v}_{i, j}^{t} \right)}{\left( \Delta x \right)^{2}} \right]$$

$$-\lambda_{Th}\left[ \frac{\left( u_{i+1, j}^{t}-u_{i-1, j}^{t} \right)}{2\Delta x}\times\frac{\left( v_{i+1, j}^{t}-v_{i-1, j}^{t} \right)}{2\Delta x}+\frac{\left( u_{i, j+1}^{t}-u_{i, j-1}^{t} \right)}{2\Delta x}\times\frac{\left( v_{i, j+1}^{t}-v_{i, j-1}^{t} \right)}{2\Delta x}+u_{i,j}^{t}\frac{\left( v_{i+1, j}^{t}+v_{i+1, j}^{t}+v_{i, j+1}^{t}+v_{i, j-1}^{t}-{4v}_{i, j}^{t} \right)}{\left( \Delta x \right)^{2}} \right]$$

$$-\lambda_{de}u_{i,j}^{t}v_{i,j}^{t}$$

Rearranging it, we obtained:

$$u_{i,j}^{t+1}=\left( 1-4r \right)u_{i,j}^{t}+r\left( u_{i+1,j}^{t}+u_{i-1,j}^{t}+u_{i,j+1}^{t}+u_{i,j-1}^{t} \right)$$

$$+s\left[ u_{i,j}^{t}\left( v_{i+1, j}^{t}+v_{i+1, j}^{t}+v_{i, j+1}^{t}+v_{i, j-1}^{t}-{4v}_{i, j}^{t} \right)+\left( u_{i+1, j}^{t}-u_{i-1, j}^{t} \right)\left( v_{i+1, j}^{t}-v_{i-1, j}^{t} \right)+\left( u_{i, j+1}^{t}-u_{i, j-1}^{t} \right)\left( v_{i, j+1}^{t}-v_{i, j-1}^{t} \right) \right]$$

$$-\alpha u_{i,j}^{t}+\beta$$

$$r=D_{u}\Delta t/\left( \Delta x \right)^{2}$$

$$s=-\left( \lambda_{TC}+\lambda_{Th} \right){\Delta t}/{\left( 2\Delta x \right)^{2}}$$

$$\alpha=-\left( \lambda_{A}+\lambda_{de}v_{i,j}^{t} \right)\Delta t$$

$$\beta={(\lambda}_{P}-\lambda_{C})v_{i,j}^{t}\Delta t$$
